# Ultra-Low-Dose Inhalation of Melphalan as an Additional Treatment for COVID-19-Associated Pneumonia

**DOI:** 10.3390/jcm14072149

**Published:** 2025-03-21

**Authors:** Evgeny Sinitsyn, Alexandra Zykova, Roman Shamin, Anna Rvacheva, Anna Bogatyreva, Elena Yarovaya, Svetlana Kuzyakina, Vladimir Kutsenko, Tatyana Shapovalenko, Antonina Stadnikova, Kirill Zykov

**Affiliations:** 1 Federal State Budgetary Institution “Pulmonology Scientific Research Institute” Under Federal Medical and Biological Agency of Russian Federation, 115682 Moscow, Russia; arvatcheva@mail.ru; 2 Federal State Budgetary Educational Institution of Higher Education “Russian University of Medicine” of the Ministry of Healthcare of the Russian Federation, 127006 Moscow, Russia; alexandra-z@mail.ru (A.Z.); ab1631611@gmail.com (A.B.); 3 LLS <Klinika Bud Zdorov>, 119146 Moscow, Russia; 4 Department of Mathematics and IT, Moscow Metropolitan Governance Yury Luzhkov University, 125032 Moscow, Russia; roman@shamin.ru; 5 Federal State Budgetary Educational Institution of Higher Education, M. V. Lomonosov Moscow State University, 119234 Moscow, Russia; yarovaya@lmech.math.msu.su; 6 Federal State Budgetary Institution “National Medical Research Center for Therapy and Preventive Medicine” of the Ministry of Health of the Russian Federation, 101000 Moscow, Russia; svetlanakuzyak@gmail.com (S.K.); vlakutsenko@ya.ru (V.K.); 7 State Budgetary Healthcare Institution of Moscow Region Children’s Clinical Hospital Named After L.M. Roshal, 143407 Krasnogorsk, Russia; mz_pdls_modb@mosreg.ru; 8 Moscow State Budgetary Healthcare Institution «Children’s City Clinical Hospital Named After Z.A. Bashlyaeva, Moscow City Health Department», 125373 Moscow, Russia; tonya-st@yandex.ru

**Keywords:** COVID-19, treatment, melphalan, inhalations, inflammation

## Abstract

**Background/Objectives:** Effective anti-inflammatory treatment for COVID-19 is necessary. It was shown that ultra-low doses (100-fold lower than therapeutic ones) of alkylating drug melphalan (MEL) interact with cytokine cell receptors without DNA damage. A method of treating severe COVID-19 with ultra-low doses of MEL inhalations was proposed. The objective was to study the efficacy and safety of MEL inhalations for COVID-19 pneumonia treatment. **Methods:** An open-label comparative study (NCT04380376) with 120 patients divided into two groups was conducted. The control group (CG) received standard treatment, and the melphalan group (MG) also received seven daily 0.1 mg MEL inhalations. Changes in clinical improvement, inflammatory markers, and CT lung scan data were primary and secondary endpoints. **Results:** Patients in the MG showed significantly better clinical outcomes compared to the CG, with improvements in dyspnea according to the WHO Ordinal Scale of Clinical Improvement and the modified Borg Scale, CT scans, and inflammatory markers. No adverse effects (including irritant and bronchoconstrictor effects) possibly related to MEL were reported. **Conclusions:** This study demonstrated the efficacy of incorporating ultra-low-dose MEL inhalations into the therapeutic regimen for patients with COVID-19-associated pneumonia. This conclusion is supported by a statistically significant improvement in clinical outcomes, as assessed by the OSCI, a more rapid reduction in the severity of dyspnea, and a marked anti-inflammatory effect, evidenced by a faster decline in C-reactive protein levels. No adverse effects were observed with the proposed treatment method. Further large-scale randomized clinical trials are warranted to validate these findings and to evaluate the potential for the implementation of ultra-low-dose MEL inhalation in clinical practice.

## 1. Introduction

The urgency of the COVID-19 problem is clear. According to the WHO, >777 million patients with confirmed SARS-CoV-2 have been recorded worldwide, with >7 million deaths [1]. About 21 million cases of COVID-19 have been registered in the Russian Federation, of which almost 400,000 cases (1.9%) were fatal [2].

At present, there are no universal approaches to the treatment of COVID-19. In all the countries of the world, many constantly changing recommendations are issued, containing an increasing number of drugs and various schemes for their use, despite the fact that the level of evidence of those recommendations is not always sufficient.

Specifically, this is due to the complexity of conducting high-quality prolonged randomized clinical trials in conditions of mass sanitary losses. Practitioners are forced to make decisions in an “infodemic” environment, that is, an environment with an overabundance of information. These situations require a search for new highly effective methods of treatment and high-quality prospective studies with an assessment of their effectiveness and safety.

Pathogenetic therapy, along with etiotropic and symptomatic therapies, is an important part of COVID-19 treatment. It is aimed at reducing the hyperergic immune response induced by SARS-CoV-2. At present, systemic glucocorticosteroids, the inhibitors of the IL-6 receptor, the inhibitors of IL-6, IL-17, and IL-1, and JAK kinase inhibitors are used in clinical practice for this purpose [3]. The possibility of using anti-CD20 Abs [4] and TNF-α blockers [5] is being considered. In a meta-analysis of data from cancer patients who underwent COVID-19 and received chemotherapy, it was shown that the previous use of type 2 topoisomerase inhibitors and alkylating drugs was associated with a decrease in mortality, which was regarded as a possible protective effect of these drugs in patients with COVID-19 that required further study [6]. Other studies have described the isolated use of full doses of alkylating drugs (e.g., cyclophosphamide) for COVID-19 treatment [7,8]. These drugs in therapeutic doses are known to have pronounced side effects due to their cytotoxic effect, which imposes serious restrictions on their widespread use in common practice.

Previously, experiments had shown that with a consistent decrease in the dose of alkylating drugs to ultra-low levels (100-fold lower than cytostatic levels), the number of targets for alkylation decreases, and the drug converts from a cytostatic agent into a cell growth modifier [9]. The anti-inflammatory effect is presumably mediated by selective blockade of the IL-2 receptor β-chain (IL-2Rβ, CD122) on the surface of effector cells. [10]. In addition, alkylating agents can interfere with signal transmission through the receptor for TNF type 1 at ultra-low concentrations, thereby providing a protective effect on a fibroblastoid cell line from the cytotoxic effect of TNF-α [11]. Thus, alkylating drugs at ultra-low doses are not cytotoxic but are instead immunoregulatory compounds.

Based on available experimental data and previous positive clinical experience, melphalan (MEL) was chosen as the drug for investigation, initially being an active compound, unlike cyclophosphamide. In preclinical studies in vivo, ultra-low doses of MEL effectively slowed down the development of dextran sulfate sodium (DSS)-induced colitis in mice, providing anti-inflammatory and regenerative effects on colon mucosal cells, which significantly increased the survival of laboratory animals [12].

It was previously shown that 46 patients with severe bronchial asthma treated with MEL showed a decrease in dyspnea and cough, an increase in exercise tolerance, a decrease in the frequency of use of inhaled short-acting β2-agonists, and a significant decrease in the number of exacerbations. There was also an improvement in spirometry results, and during fibrobronchoscopy, significant decreases in hyperemia, edema, and bronchial secretion viscosity were found. According to results from electron microscopy, there was a positive dynamic of morphological changes in the mucous membrane of the bronchial tree. The proven absence of a local irritating effect and the absence of negative effects on laboratory parameters of the drug in ultra-low doses are noteworthy [13,14].

In this regard, given the previously demonstrated noncytotoxic anti-inflammatory effect, we made an assumption about the potential effectiveness of ultra-low doses of MEL in COVID-19-associated pneumonia. The aim of the present study was to evaluate the efficacy and safety of an ultra-low dose of inhaled MEL in hospitalized patients with COVID-19-associated pneumonia.

## 2. Materials and Methods

This study was registered on ClinicalTrials.gov (NCT04380376) and was conducted at Clinical Hospital #1 MEDSI (Moscow, Russian Federation) from May to December 2020.

Study design: A single-center, prospective, open-label, retrospective-controlled, central-blinded comparative study was designed. This study, by decision of a medical commission, included hospitalized adult patients with an established diagnosis of moderate or severe COVID-19-associated pneumonia, who, in the opinion of their attending physician, tended to have a worsening clinical condition. These patients received MEL inhalations (within ~10 min) at an estimated delivered dose of 0.1 mg/d (0.05 mg/mL) via a compressor nebulizer once a day for 7 d during standard therapy. The CG included patients who received standard therapy. They were retrospectively selected by an independent expert using software for comparative analysis according to the case–control principle; that is, pairs of patients were selected who were comparable in clinical condition at the time of admission to the hospital. Standard therapy was carried out in accordance with the current version of the Interim Clinical Guidelines of the Ministry of Health of the Russian Federation at the time of hospitalization [15,16,17,18].

In accordance with the protocol, 120 patients (60 in the MG and 60 in the CG) were included in the study. Inclusion criteria were as follows: signed informed consent, age ≥ 18 years, an established diagnosis of COVID-19 with pulmonary lesions characteristics for COVID-19 infection on CT scans [15], and the patient had at least one of the following symptoms: fever > 38 °C, cough, dyspnea, tachypnea, or hypoxemia (oxygen saturation (SpO2) < 95% when breathing atmospheric air).

The criteria for non-inclusion and exclusion were as follows: withdrawal of informed consent by the patient, unwillingness of the patient to follow the instructions of the research staff regarding the requirements of the study protocol, identification of noncompliance with the inclusion criteria, an AE that, in the opinion of the investigator, could become unsafe for the patient, termination of the study at the request of regulatory authorities, and, for female patients, a planned or ongoing pregnancy and/or breastfeeding.

Patient selection method for the CG: The development of this algorithm was carried out on the basis of the Institute of Advanced Technologies and Industrial Programming of the Federal State Budgetary Educational Institution of Higher Education “MIREA–Russian Technological University” (Moscow, Russian Federation). The method of selecting “close” patients was carried out using an electronic database of case histories. For each MG patient, a list of the closest patients was selected, indicating the proximity measure in dimensionless values. The operation algorithm of the technique included the construction of a tabular database, the normalization of patient parameters, the construction of a metric space of patients, the selection of close patients, and the manual verification of the calculation results. The total number of COVID-19 patients in the database was 2000.

The tabular database was constructed as follows: each medical history from a text format was converted into tabular information, which included the number of the medical history, the year of birth of the patient, the dates of hospitalization and discharge, the number of days from the onset of the disease, the fact of being in the intensive care unit, known concomitant diseases at the time of hospitalization, the results of clinical analyses, and lung CT scan data. To ensure confidentiality, the medical records did not contain personal data (e.g., full names, addresses, and phone numbers), and these data were not used in further work. To implement this step, textual information processing methods (data mining) were used.

Subsequently, the parameters of patients were normalized. All patient parameters were normalized to numbers from 0 to 1 so that the minimum value of the parameter corresponded to 0 and the maximum to 1. This normalization additionally makes the values of the parameters “dimensionless”. A linear normalization procedure was used. As a result of the normalization procedure, each patient was represented by an n-dimensional numerical vector, with components from 0 to 1. The dimension of n was determined by the parameters that were used to describe the patients.

This was followed by the construction of the metric space of patients. To do this, the set of patients was represented as a metric space; that is, for each pair of patients, a numerical value was determined, being a metric that evaluated the measure of the “proximity of these patients.” Let X and Y be two patients. After normalization, these were two numeric vectors, X = (x1, x2, …, xn) and Y = (y1, y2, …, yn). The metric between them was calculated as follows: ϱ(X, Y) = α1|x1 − y1| + α2|x2 − y2| + … + αn|xn − yn|, where α1, α2, …, and αn were positive weight coefficients that determined the importance of one or another parameter. These coefficients were chosen by an expert. However, as a result of normalization at the second step, the results of the algorithm were also satisfactory in the case of α1 = α2 = … = αn = 1.

Furthermore, for each patient Z, a list of the closest patients was built as follows: for each patient from the database of patient X, the metric ϱ(X, Y) was built, and all patients were ordered in descending order of the values of this metric. At the same time, patients who had previously received MEL were excluded from the comparison procedure. Each pair of close patients was verified manually by an independent expert, using all available information about the patients.

The effectiveness of treatment was assessed by changes according to the WHO Ordinal Scale of Clinical Improvement (OSCI) [19] and the dynamics of dyspnea severity according to the modified Borg Scale (mBS) from 0 to 10 points [20], completed by the patients of both groups. For both scales, a 1-point change in category was considered clinically significant. In addition, the proportion of patients with clinical improvements on days 7, 14, and 28 (or the day of discharge, whichever came first) from the start of MEL therapy or the corresponding day in the CG was determined. For this purpose, the following were recorded: SpO2 in the atmospheric air by pulse oximetry, body temperature in the armpit using a Galinstan thermometer, RR, and cough intensity, assessed by the doctor during the collection of complaints according to the following gradations: 0, no; 1, light; 2, moderate; 3, severe. The following criteria for achieving clinical improvement were considered: body temperature < 37 °C, respiratory rate (RR) ≤ 24/min in room air, and SpO2 > 94% in ambient air, and for cough, an absence or decrease in cough intensity (0 or 1 points).

Safety assessment was carried out by registering all adverse events (AEs) for the MG, as well as on the basis of dynamics assessment for clinical, biochemical, and immunological blood tests at the start of treatment in the MG and the corresponding day in the CG, on days 7, 14, and 28 (or the day of discharge, whichever came first). In addition, the MG patients underwent daily pulse oximetry before and immediately after inhalation to exclude the irritant and bronchoconstrictive effects of the drug.

AEs that occurred during hospitalization for the CG were manually selected from medical diaries about the condition of patients and discharge records.

CT scans of the lungs were performed on the devices LightSpeed VCT (General Electric) and Brilliance iCT Elite (Philips). Scanning was performed in an axial projection with a slice thickness of 0.625–1 mm at mA 350–400 and 120 kV, followed by iterative reconstruction. The percentage of lung damage was calculated semiautomatically by the Philips Portal v11 chronic obstructive pulmonary disease lung density computer evaluation program. The dynamics of lesion volume on CT scans were assessed from the start day of inhalations in the MG and from the corresponding day in the CG, and also at the time of discharge from the hospital.

Statistical analysis: Because this study did not perform a formal hypothesis test and was a pilot study, the power of estimation was not calculated for the required number of patients in each treatment group. We judged outcomes statistically significant when the *p* value was <0.05. Non-skewed continuous variables are presented as mean (SD). Skewed parameters are presented as median [Q1; Q3]. Categorical variables are presented as n (%). We used the Mann–Whitney U test to compare continuous variables and the two-sided Fisher’s exact test to compare categorical variables. To compare the parameter dynamics of treatment in the two groups, the method of repeated measures (ANOVA) was used. Measurement comparisons in this case were carried out taking into account multiple comparisons using the Holm–Bonferroni correction. All statistical analyses were performed using R (version 3.5.1).

## 3. Results

The melphalan group (MG) included 38 men and 22 women, and the control group (CG) included 40 men and 20 women. The average age in the MG was 53.4 ± 13.5 y. In the CG, the average age was 57.0 ± 13.3 y.

The average number of days before hospitalization from the moment of the appearance of the first clinical symptoms of the disease in the MG was 8.4 ± 3 d; in the CG, it was 8.7 ± 4 d.

At the start of MEL therapy, the MG patients had significantly worse clinical conditions in terms of the OSCI, mBS, and SpO2 level. Initially, complaints of cough were presented by patients from the MG in a greater number of cases (68.3%) than in the CG (53.3%). At the same time, the intensity of cough was significantly higher in the MG than in the CG, that is, 2 [0, 2] and 1 [0, 2] points, respectively. There were no significant differences between the groups in terms of body temperature and RR.

At the start of therapy, MG patients had larger lung lesion volumes on CT scans, which did not reach a significant difference when compared with those in the CG. There were no differences in the severity of the disease in both groups (see Table 1).

A larger percentage of cases with positive reverse transcriptase–PCR (RT-PCR) to SARS-CoV-2 was detected in the MG (43.3 versus 28.3% in the CG), but the difference did not reach statistical significance.

According to laboratory parameters, a significantly higher level of C-reactive protein (CRP; 76.3 [29.4, 112.3] mg/L) was observed in the MG compared to the CG (38.4 [17.5, 95.8] mg/L). There was a significant difference in the level of platelets, which was lower in the MG compared to the CG (180.0 [160.0, 210.0] and 204.0 [169, 277] × 10^9^/L, respectively).

The standard therapy drugs for COVID-19 are presented in Table 1. There were no significant differences between the groups in the prescription of etiotropic therapy drugs. Among the means of pathogenetic treatment, macrolides (95 and 71.7%), inhibitors of JAK kinases (36.7 and 5%), and antioxidants (95 and 58.3%) were prescribed significantly more often in the MG than in the CG. In turn, the inhibitors of IL and their receptors and systemic glucocorticosteroids were more often prescribed in the CG than in the MG (11.7% and 6.7%; 15% and 10%, respectively). Of the symptomatic therapy drugs, probiotics (93.3 and 70%) and antihistamines (53.3 and 33.3%) were prescribed more often in the MG than in the CG.

The mean hospital stay did not differ significantly between the groups and was 13.3 ± 7 d in the MG and 12.9 ± 6 d in the CG.

**Figure 1:**
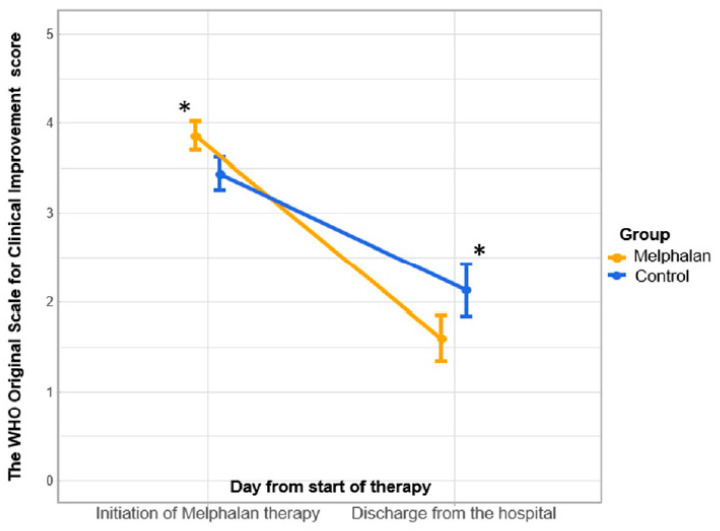
WHO scale (OSCI) dynamics for the mean. *—*p* < 0.05. Vertical bars denote 0.95 confidence intervals for the mean. Initially, the MG had significantly worse indicators. By the time of discharge, the indicators in the MG were significantly better. A difference in the dynamics of the indicator between groups was revealed.

With a significantly more severe initial clinical condition of MG patients at the beginning of MEL therapy, a positive trend in OSCI was revealed in both groups by the time of discharge from the hospital: from 4.0 [3.0, 4.0] points to 1.0 [1.0, 2.0] points in the MG (*p* < 0.05) and from 3.0 [3.0, 4.0] points to 2.0 [2.0, 2.0] points in the CG (*p* < 0.05). At the same time, a significant difference was recorded between the MG and the CG (1.0 [1.0, 2.0] points and 2.0 [2.0, 2.0] points, respectively) (*p* < 0.05) at the time of discharge (shown in Figure 1).

**Figure 2:**
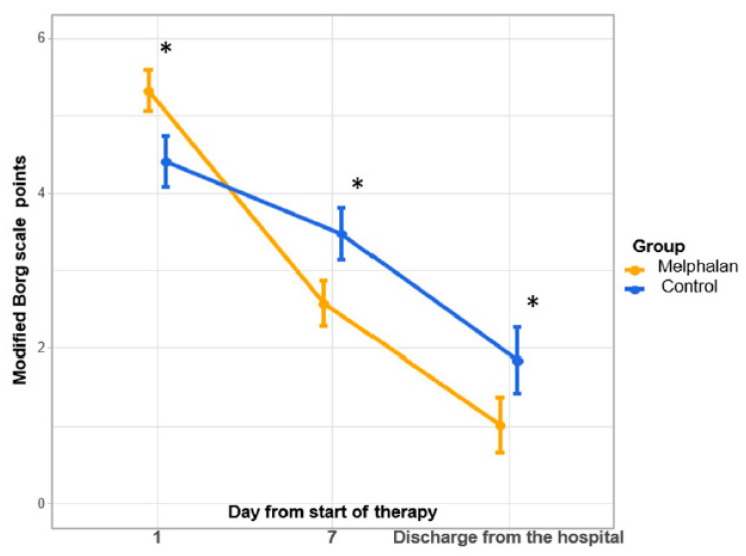
Borg Scale dynamics for the mean. *—*p* < 0.05. Vertical bars denote 0.95 confidence intervals for the mean. Initially, dyspnea was significantly more pronounced in the MG. By the 7th day of treatment in MG, dyspnea is significantly less present, as well as by discharge.

With initially more pronounced dyspnea in MG patients according to the mBS, by the seventh day of therapy, a significant decrease in its intensity was noted in both groups, that is, from 5.0 [4.0, 6.0] points to 3.0 [2.0, 3.0] points in the MG and from 4.0 [3.0, 5.0] points to 3.0 [3.0, 4.0] points in the CG, respectively, whereas in the MG, the intensity of dyspnea became significantly lower than in the CG (3.0 [2.0, 3.0] points and 3.0 [3.0, 4.0] points, respectively). By the time of discharge from the hospital, the intensity of dyspnea continued to significantly decrease in both groups, although there was also a more pronounced decrease in dyspnea in the MG compared to the CG (*p* < 0.05) (shown in Figure 2).

When analyzing the SpO2 parameters in the study groups, we found that by the time MEL therapy was started in the MG, the SpO2 values were significantly lower than in the CG, that is, 93 [90, 96]% and 96 [93, 97]%, respectively. By the seventh day of therapy and by the day of discharge from the hospital, the SpO2 values in both groups increased significantly, although the difference between the groups was leveled.

The intensity of cough by the start of MEL therapy was significantly higher in the MG than in the CG, that is, 2 [0, 2] points and 1 [0, 1] point, respectively. By the seventh day of therapy, the cough intensity significantly decreased in both groups, although there were no significant differences between the groups.

The parameters of body temperature and RR in the patients of both groups did not differ initially. By the seventh day of therapy and by the day of discharge in both groups, the indicators improved, with no statistically significant difference between the groups.

By the seventh day of therapy in the MG, 65% of patients achieved clinical improvement against 75% in the CG, although there was no significant difference between the groups. By the time of discharge in both groups, this indicator significantly increased and amounted to 95% in both groups, with no significant difference between the groups having been found.

The dynamics of lesion volume on CT scans were characterized by a statistically significant improvement in both groups, with no significant difference between the groups both at the beginning of therapy and by the time of discharge from the hospital. At the same time, the dynamics of improvement in terms of the lesion volume were significantly more pronounced in the MG (5.05 [2, 17.3]%) than in the CG (4.35 [0, 9.95]%).

Table 2 presents the dynamics data of laboratory tests. When analyzing the dynamics of clinical blood test indicators, there were no significant changes in any laboratory parameters in either group. Laboratory parameters normalized until the day of discharge in most patients, with slight differences between groups; most were within normal laboratory ranges.

Three serious adverse events (SAEs) were registered in the MG, while two consecutive SAEs were registered in one patient (intubation due to acute respiratory failure, and then death). Another patient from the MG had one SAE (intubation due to acute respiratory failure with subsequent clinical improvement). None of the reported SAEs were found to be associated with treatment with MEL.

Five SAEs were registered in the control group. Two patients had two consecutive SAEs (intubation due to acute respiratory failure, and then death), and one had one SAE (intubation due to acute respiratory failure with subsequent clinical improvement).

All AEs related to laboratory findings were grades 1–2. No AE was considered related to MEL in the MG. During the trial, we collected all AEs in the MG and manually selected information on AEs from medical diaries and discharge records from the CG.

During hospitalization, four (6.6%) patients from the MG and six (10%) patients from the CG reported a temporary cough increase (grade 2), which was resolved by discharge day. Three (5%) patients from the CG and one (1.6%) patient from the MG reported deep vein thrombosis (detected via ultrasound assessments), with all having been grade 2; in all cases, patients received symptomatic treatment. One (1.6%) patient in the MG had grade 3 nasal bleeding. The patient underwent aortal value replacement in 2010 and was put on warfarin treatment (prothrombin time, 60.4 s; international normalization ratio, 6.13; platelet level, 219 × 10^9^/L). One (1.6%) patient in the CG reported acute clostridium colitis (grade 3). The patient was treated with ceftriaxone and azithromycin before hospitalization, and with clarithromycin and vancomycin during hospitalization. Two (3.3%) patients from the CG reported grade 2 anemia.

**Figure 3:**
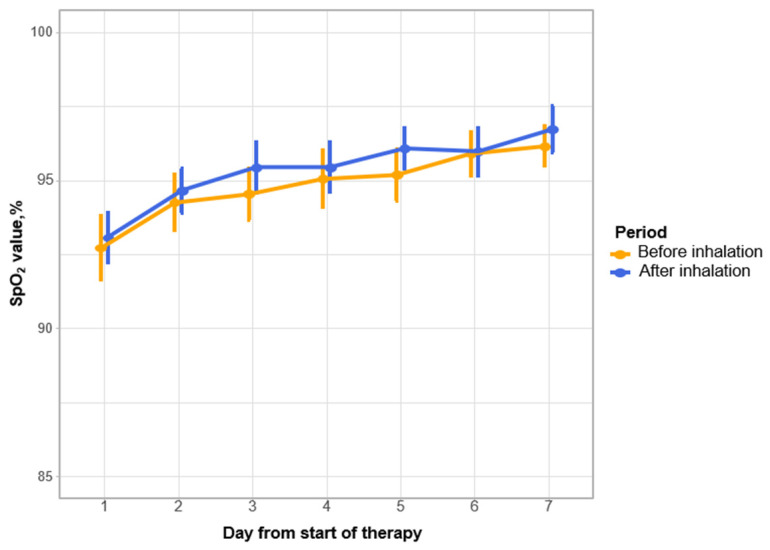
SpO2 value dynamics for the mean in MG. Vertical bars denote 0.95 confidence intervals for the mean. There are no differences at any of the stages of observation between the groups.

At the time of inclusion in this study, mechanical ventilation was performed in one (1.6%) MG patient and in two (3.3%) CG patients. By the seventh day of therapy, one (1.6%) case of intubation was registered in the MG and two cases in the CG. At subsequent points of observation, intubation was not registered in either group. By the end of the hospital stay in the MG, one (1.6%) patient was extubated and one (1.6%) patient died. In the CG, one (1.6%) patient was extubated and two (3.3%) patients died. Thus, by the end of the hospital stay, in the MG, 96.7% of patients did not undergo mechanical ventilation versus 96% in the CG. No AEs possibly related to MEL inhalation were reported in the trial. All inhalations were well tolerated, no specific signs/symptoms were reported, and no patients were withdrawn from inhalation treatment. Against the background of therapy in the MG, there was no decrease in the SpO2 level immediately after inhalation administration of MEL, and despite that, oxygen flow during inhalation remained unchanged (shown in Figure 3).

## 4. Discussion

One of the main threats in COVID-19 is an excessive immune response, which includes the hyperactivation of effector cells with the uncontrolled release of cytokines and other inflammatory factors. Such a scenario can lead to massive lung damage, the rapid progression of respiratory failure, the development of acute respiratory distress syndrome, multiple organ failure, and death. In this regard, the search for effective and safe methods of pathogenetic treatment aimed at suppressing the hyperimmune response remains important.

Throughout the COVID-19 pandemic, numerous attempts have been made to use various anti-inflammatory agents targeting the disease’s pathogenic mechanisms. These efforts have included the repurposing of existing drugs approved for other indications, such as famotidine, metformin, ivermectin, and fluvoxamine, among others [21,22].

Currently, anti-inflammatory drugs developed for the treatment of other diseases are widely used for this purpose (e.g., glucocorticoids, inhibitors of various interleukins and their receptors, and inhibitors of JAK kinases). The question of possibly using alkylating drugs in COVID-19 has been discussed, as it has been shown that the previous use of these compounds by cancer patients was associated with a decrease in mortality [6]. Single attempts to use full therapeutic doses of cyclophosphamide for the treatment of COVID-19 have been described [7,8]. The most important limitations of the use of these compounds are the side effects due to cytostatic action. One of the possible ways to reduce the risk of side effects is the use of inhalations of ultra-low (100-fold lower than therapeutic) doses of alkylating drugs, in particular MEL. The inhalation route of MEL administration is possible because it is an initially active compound (unlike cyclophosphamide) that does not require prior contact with cytochrome P-450. This method allows the delivery of the drug directly to the target organ, which in turn makes it possible to reduce the total dose of the drug and reduce the systemic effect, and, as a result, reduce the risk of side effects [23]. Previously, it was shown that at ultra-low doses, MEL does not have a local irritating effect and is able to interrupt signaling by blocking the IL-2 receptor β-chain on the surface of effector cells and the TNF-α/TNF receptor-associated death domain (TRADD) chain [12]. At the same time, low MEL doses exhibit not cytostatic but immunocorrective anti-inflammatory properties, which was previously demonstrated in the treatment of patients with severe bronchial asthma [13,14]. These properties suggested the possibility of its use in the treatment of COVID-19-associated pneumonia.

In the course of this study, MEL demonstrated its effectiveness in two primary endpoints: a more pronounced clinical effect on the OSCI and a more rapid decrease in the intensity of dyspnea on the mBS were noted, despite the fact that initially, these indicators in the MG were significantly worse than in the CG. This confirms the clinical efficacy of ultra-low-dose inhaled MEL for the treatment of COVID-19-associated pneumonia.

Analysis of the standard COVID-19 therapy revealed differences between the groups in several areas: JAK kinase inhibitors, macrolides, antioxidants, probiotics, and H1-antihistamines. The ambiguity of available data on the efficacy of antioxidants [24,25,26,27], the insufficient evidence base for the efficacy of probiotics [28] in COVID-19, and the complete absence of such studies for H1-antihistamines allow us to disregard the identified differences. It should be noted that JAK kinase inhibitors were more frequently prescribed in the MG, while systemic glucocorticosteroids and anti-IL6 agents were more commonly prescribed in the CG. To assess the potential impact of the frequency of JAK kinase inhibitor prescriptions on the study outcomes, a sub-analysis of the total frequency of immunosuppressant prescriptions (systemic glucocorticosteroids, JAK kinase inhibitors, and IL and IL receptor blockers) was conducted in both groups. This sub-analysis did not reveal any significant differences between the prescription of any of the immunosuppressants in the study groups, which substantially reduces the potential influence of the disparity in JAK kinase inhibitor prescriptions on the clinical outcomes of this study. This study observed a higher frequency of macrolide prescriptions in the main group. However, it is important to note that macrolides have demonstrated an impact on COVID-19 outcomes only when administered early in the disease course [29]. In the present study, macrolide therapy was initiated at later stages of the disease, on average on the 8th day from symptom onset, which significantly diminishes the potential influence of macrolide administration on the obtained clinical outcomes [30].

Initially, the level of CRP in the MG was significantly higher than in the CG. The leveling of the statistically significant difference between the groups by the seventh day of therapy against the background of a significant decrease in the level of CRP in both groups indicates the anti-inflammatory effect of inhalations of ultra-low doses of MEL.

There was no statistically significant difference in the proportion of patients who achieved clinical improvement in the MG and CG (according to body temperature, RR, SpO2, and cough intensity). This is probably due to the fact that at the time of enrollment in the study, patients were already in treatment. Thus, the active use of nonsteroidal anti-inflammatory drugs for the relief of fever in all patients led to the fact that the values of body temperature at the time of inclusion in the study did not go beyond normal values. The RR in both groups by the time of inclusion in the study was also compensated by inspiratory support and ongoing anti-inflammatory therapy, which led to a decrease in the manifestations of general infectious intoxication syndrome. However, SpO2 and the frequency and intensity of cough, which were significantly worse in the MG at the time of inclusion in this study, improved so much by the seventh day of MEL therapy that a significant difference with the CG was leveled.

One of the most important results of the present study is the fact that when using the study drug, no treatment-emergent AEs related to MEL inhalation were registered, and there was no local irritant or bronchoconstrictive effect, which was confirmed by the absence of a decrease in saturation on the background of inhalations. No significant dynamics of the blood test parameters were revealed, which indicates the absence of negative systemic effects of inhaled ultra-low doses of MEL, in contrast to its therapeutic doses administered orally or parenterally. There were no local or systemic allergic reactions, bronchospasms, or interstitial pneumonitis from the respiratory organs. From the hematopoietic system side, we did not find increases in myelodepression (leukopenia, thrombocytopenia, and anemia), bleeding and hemorrhage, vasculitis, veno-occlusive lesions, and signs of gastrointestinal lesions such as stomatitis, nausea, vomiting, esophagitis, gastrointestinal bleeding, or gastric and duodenal ulcers. All of the above findings verify the safety of the proposed method of treatment.

In the course of our work, it was found that there was a lower percentage of intubations in the MG, although it did not reach a significant difference between the groups, which is explained by the small sample of patients.

It is noteworthy that none of the MG patients withdrew from this study. It is not possible to reliably assess the effect of the method on mortality due to the small number of events, since one lethal outcome was registered in the MG and two occurred in the CG.

Taking into account that the work was carried out in a real inpatient clinical practice, this study included patients with a clinical diagnosis of COVID-19, both with laboratory-confirmed SARS-CoV-2 and with clinical and radiological signs of COVID-19 without RT-PCR confirmation. It has been shown that an RT-PCR test can give a negative result when the amount of viral genome in the sample is insufficient or when the viral replication period in the upper respiratory tract is missed [31]. Although the incubation period of COVID-19 is estimated at 5 d, negative results can be obtained as early as 7 d after infection [32]. In this study, patients were admitted to the hospital on average on the eighth day from the onset of clinical symptoms, which inevitably led to a low percentage of positive RT-PCR diagnostics at the time of hospitalization. Thus, the inclusion of only RT-PCR-positive patients in the study would not allow extrapolating the results obtained to the general sample of patients with COVID-19-associated pneumonia in real inpatient clinical practice.

We would like to emphasize that the pilot nature of our study, which included 120 patients, does not provide sufficient statistical power to draw definitive conclusions regarding the safety and efficacy of the proposed method. Such judgments and broader generalizations would require the organization of a larger-scale, more comprehensive investigation. We acknowledge the importance of further research to validate and expand upon our findings.

## 5. Conclusions

The data obtained indicate the efficacy of treating patients with COVID-19-associated pneumonia using inhalations of ultra-low doses of MEL as part of therapy, which is supported by a significantly more pronounced clinical effect according to the OSCI, a faster decrease in the intensity of dyspnea, and an anti-inflammatory effect, which was expressed in a faster decrease in the CRP level. No side effects were found when using the proposed method. A prospective clinical study in a larger sample of patients is needed to address the issue of the feasibility of the widespread use of ultra-low-dose inhalations of the alkylating drug MEL.

## Figures and Tables

**Table 1:** Baseline characteristics of patients.

Parameter	MG (n = 60)	CG (n = 60)
Descriptive statistics of the study population ^A^		
Male, *n* (%)	38 (63.3)	40 (66.7)
Female, *n* (%)	22 (36.7)	20 (33.3)
Age, y	53.4 ± 13.5	57 ± 13.3
Average days of illness before hospitalization	8.4 ± 3	8.7 ± 4
Severity distribution ^A^		
Moderate course, abs. (%)	45 (75)	50 (83.3)
Severe course, abs. (%)	15 (25)	10 (16.7)
RT-PCR ^A^		
Positive, abs. (%)	26 (43.3)	17 (28.3)
Negative, abs. (%)	34 (56.7)	43 (72.7)
Lesion volume on CT scans by groups ^B^ % (median)	28.1 [17.4, 42.0]	21.8 [16.7, 30.2]
OSCI ^B^ by groups, points (median)	4.0 [3.0, 4.0] ^C^	3.0 [3.0, 4.0]
mBS ^B^ by groups, points (median)	5 [4, 6] ^C^	4 [3, 5]
Clinical parameters ^B^		
Cough, points		
No. of patients with cough, abs. (%)	41 (68.3)	32 (53.3)
Median	2 [0, 2] ^C^	1 [0, 2]
Median body temperature, °C	36.9 [36.6, 37.5]	36.9 [36.7, 37.5]
Median RR, abs.	19 [18, 20]	19 [18, 22]
Median oxygen saturation, %	93 [91, 96] ^C^	96 [93, 97]
COVID-19 therapy drugs, abs. (%)		
Etiotropic therapy		
Antivirals (nucleotide inhibitors of viral RNA polymerase)	56 (96.7)	48 (90)
Pathogenetic therapy		
Macrolides ^C^	57 (95)	43 (71.7)
Anticoagulants	60 (100)	60 (100)
Inhibitors of ILs and their receptors	4 (6.7)	7 (11.7)
Antioxidants ^C^	57 (95)	35 (58.3)
JAK kinase inhibitors ^C^	22 (36.7)	3(5)
Glucocorticosteroids	6 (10)	9 (15)
Symptomatic therapy		
Antibacterial therapy (except macrolides)	52 (86.7)	41 (68.3)
Nonsteroidal anti-inflammatory drugs	60 (100)	60 (100)
Gastroprotectors	60 (100)	59 (98.3)
Probiotics ^C^	56 (93.3)	42 (70)
Expectorants	3 (5)	6 (10)
Nootropic drugs	5 (8.3)	8 (13.3)
Antidepressants	8 (13.3)	13 (21.7)
Antihistamines ^C^	32 (53.3)	20 (33.3)
Antifungal drugs	8 (13.3)	6 (10)
Hepatoprotectors	12 (20)	9 (15)

^A^ at the time of hospitalization. ^B^ at the time of initiation of MEL therapy. ^C^ *p* < 0.05. abs, absolute value.

**Table 2:** The dynamics of laboratory data.

Parameter (Reference Values)	Melphalan Group			Control Group		
	Initiation of Treatment (Day 1)	End of Treatment (Day 7)	Discharge from Hospital	Day Corresponding to Start of Treatment in the MG	Day Corresponding to End of Treatment in the MG	Discharge from Hospital
Leukocytes × 10^9^/L (N 4–8.8)	5.1 [4.2, 6.7]	5.7 [4.3, 6.9]	5.6 [4.6, 7.2]	5.1 [4.1, 6.4]	5.4 [4.7, 6.4]	5.8 [4.8, 6.6]
Neutrophils × 10^9^/L (N 1.56–6.13)	3.29 [2.36, 4.66]	3.21 [2.38, 4.47]	3.03 [2.26, 4.18]	3.69 [2.46, 4.90]	2.82 [2.27, 3.78]	3.02 [2.46, 3.61]
Lymphocytes × 10^9^/L (N 1.18–3.74)	1.0 [0.77, 1.31] ^a^	1.3 [0.9, 1.6] ^b^	1.6 [1.3, 2.1]	1.0 [0.8, 1.4] ^a^	1.5 [1.1, 1.8] ^b^	1.6 [1.3, 2.0]
Platelets × 10^9^/L (N 120–380)	180 [160, 210] ^a,c^	255 [188, 370] ^b^	346 [280.5, 410.5]	204.0 [169, 277] ^a^	286.0 [241, 368]	310.5 [252, 369]
Creatinine, µmol/L (N 58–96)	92 [78, 105.5] ^a^	80.5 [69, 97.5]	83.5 [70, 92.5]	93.5 [73.5, 105.5] ^a^	78.0 [71, 91.5]	80 [70.5, 97]
Urea, mmol/L (N 1.8–6.4)	4.5 [3.8, 5.8]	4.3 [3.3, 5.3]	4.3 [3.3, 5.1] **^c^**	5.1 [3.9, 7.1]	4.9 [3.8, 5.9]	5.0 [3.9, 6.3]
AST, U/L (N 0–55)	35 [25.5, 41]	36 [26, 58]	33 [25, 50]	33 [26.5, 46.6]	39 [28, 63]	37.5 [26, 54.5]
ALT, U/L (N 0–50)	26.5 [21, 38] ^a^	38 [24, 72]	44 [24, 81]	29 [20, 41.5] ^a^	50 [31, 87]	49.5 [30, 89]
D-dimer, ng/mL (N 0–550)	502 [386, 852]	513 [366, 999]	527.5 [367, 1052]	437.5 [295.5, 794.5]	481 [259, 1005]	455 [252, 799]
CRP, mg/L (N 0–5)	76.3 [29.4, 112.3] ^a,c^	34,6 [12.3, 65.3] ^b^	7.0 [3.8, 15.3]	38.4 [17.5, 95.8] ^a^	18.0 [6.3, 43.3] ^b^	6.3 [3.1, 13.9]

Values are represented by median, [25th, 75th] percentiles. ^a^ *p* < 0.05 when comparing the start day of treatment (day 1) and the end of treatment (day 7). ^b^ *p* < 0.05 when comparing the indicators of the day of the end of treatment (day 7) and the day of discharge from the hospital. ^c^ *p* < 0.05 when comparing indicators between the study groups. N, normal value.

## Data Availability

The (primary) data that support the findings of this study are not publicly available due to their containing information that could compromise the privacy of research participants but are available from the corresponding authors Evgeny Sinitsyn and Kirill Zykov upon reasonable request.
